# Benchmarking interpretability of deep learning for predictive genomics: Recall, precision, and variability of feature attribution

**DOI:** 10.1371/journal.pcbi.1013784

**Published:** 2025-12-05

**Authors:** Justin Reynolds, Chongle Pan

**Affiliations:** School of Computer Science, Gallogly College of Engineering, University of Oklahoma, Norman, Oklahoma, United States of America; University of Western Ontario: Western University, CANADA

## Abstract

Deep neural networks can model the nonlinear architecture of polygenic traits, yet the reliability of attribution methods to identify the genetic variants driving model predictions remains uncertain. We introduce a benchmarking framework that quantifies three aspects of interpretability: attribution recall, attribution precision, and stability, and apply it to deep learning models trained on UK Biobank genotypes for standing height prediction. After quality control, feed-forward neural networks were trained on more than half a million autosomal variants from approximately 300 thousand participants and evaluated using four attribution algorithms (Saliency, Gradient SHAP, DeepLIFT, Integrated Gradients) with and without SmoothGrad noise averaging. Attribution recall was assessed using synthetic spike-in variants with known additive, dominant, recessive, and epistatic effects, enabling direct measurement of sensitivity to diverse genetic architectures. Attribution precision estimated specificity using an equal number of null decoy variants that preserved allele structure while disrupting genotype-phenotype correspondence. Stability was measured by the consistency of variant-level attributions across an ensemble of independently trained models. SmoothGrad increased average recall across effect types by approximately 0.16 at the top 1% of the most highly attributed variants and improved average precision by about 0.06 at the same threshold, while stability remained comparable with median relative standard deviations of 0.4 to 0.5 across methods. Among the evaluated attribution methods, Saliency achieved the highest composite score, indicating that its simple gradient formulation provided the best overall balance of recall, precision, and stability.

## Author summary

Understanding which genetic variants contribute most significantly to complex traits such as human height is crucial for advancing genomic research. Deep neural networks (DNNs) offer powerful predictive capabilities for these traits, but their complexity makes it challenging to interpret which genetic features drive their predictions. In this study, we developed a comprehensive framework to objectively evaluate the reliability and biological relevance of several popular interpretation methods used with DNNs. Using genotype array data from the UK Biobank, we predicted individuals’ heights and systematically assessed four widely used interpretation algorithms: Saliency, Gradient SHAP, DeepLIFT, and Integrated Gradients. We found that interpretation performance was relatively consistent across individual attribution algorithms; however, performance generally improved for the more advanced gradient-based algorithms (i.e., Gradient SHAP, DeepLIFT, and Integrated Gradients) in the presence of SmoothGrad noise averaging. These results show that gradient-based interpretation methods can effectively recover both additive and more complex non-additive genetic signals while maintaining stable and selective feature attributions.

## 1. Introduction

Deep learning (DL) has become an increasingly valuable tool for polygenic risk score (PRS) estimation by providing the capacity to model complex, nonlinear relationships and epistatic interactions among genetic variants that are often ignored by conventional PRS approaches [1,2]. Applications of deep neural networks (DNNs) to PRS estimation have demonstrated improvements in predictive accuracy across a range of traits and diseases, including Alzheimer’s disease [3,4], diabetes [4,5], inflammatory bowel disease [4], and breast cancer [4,6]. Nevertheless, these gains in predictive performance come at the cost of reduced interpretability, as the black-box nature of DNNs makes it difficult to determine which features contribute most to model predictions.

Many interpretation algorithms have been developed to address the interpretability challenge of DNNs, including Saliency [7], Gradient SHAP [8], Integrated Gradients [9], DeepLIFT [10,11], and LINA [12], among others. These algorithms perform interpretation on DNN predictions by feature attribution, which attempts to identify salient features that have a considerable impact on model predictions. However, these algorithms differ in theoretical assumptions, sensitivity to model architecture, and robustness to input changes. Such differences may lead to inconsistent attributions, particularly in high-dimensional input spaces. This challenge, while not unique to genomics or DNN-based PRS estimation, is evident in such domains where input features such as single nucleotide polymorphisms (SNPs) may be correlated and the output is determined by many input features with complex interactions. These factors complicate the task of identifying biologically meaningful signals and increase the risk that feature attributions reflect illegitimate associations rather than genuine causal effects. While several DNN-based PRS studies have incorporated interpretability methods, a common benchmarking strategy is lacking as evaluations are typically limited to qualitative visualization or heuristic checks [2,6,13], leaving the reliability and biological relevance inadequately assessed or unverified. More recent advances in statistical feature selection, such as the knockoff framework for false discovery rate control [14,15], offer correlation-aware approaches for constructing null feature sets, though their computational demands make large-scale applications challenging.

General strategies for evaluating feature importance can broadly be categorized as either objective or subjective [16]. Objective methods assess interpretability using quantifiable criteria, such as the sensitivity of feature attributions to input or model perturbations [17–21], degradation in predictive performance after removing highly ranked features [22–24], or agreement with curated datasets where feature relevance is known to some degree [20,25]. In contrast, subjective evaluations involve human-in-the-loop assessments where human judgment is used to measure the plausibility of feature attributions [20,26–28]. While these approaches have been beneficial in domains such as computer vision (CV) and natural language processing, many of them are computationally infeasible or ill-suited for genomics applications. For example, ablation-based approaches such as Remove and Retrain (ROAR) [24] require iteratively retraining the model after removal of features, which is computationally prohibitive at the genome-wide scale. Concept-oriented approaches such as TCAV [25], Self‐Explaining Neural Networks [17] and BAM [20] depend on the use of well-defined concepts such as “stripes” in a CV model trained to identify zebras in images or “hypertension” in a model trained to assess cardiovascular disease risk from electronic medical records. Such concepts would be unknown or difficult to characterize in genomic applications. Accordingly, the scale and modality differences between these methods and the present study make their inclusion as comparative approaches impractical.

In this study, we developed and evaluated a framework for benchmarking interpretation performance of feature attribution algorithms in DNN-based predictive genomics. Specifically, we introduce three metrics aimed at different facets of model interpretation. First, attribution recall is defined as the proportion of synthetic top-ranked DNN attributed features that correspond to known causal variants. Attribution recall is estimated by applying synthetic spike-in SNPs with intentional phenotype associations alongside the empirical genotype matrix, providing an objective measure of interpretation sensitivity to known phenotype-associated signals. Second, attribution precision is defined as the fraction of top-ranked salient features among all top-ranked features. We hypothesize that attribution precision can be estimated using decoy SNPs that have no real effect on the phenotype. Third, attribution consistency is defined by the stability of feature attributions across multiple independently trained models, capturing susceptibility to training differences. We hypothesize that true associations should be invariant with respect to the model training process. In this work, we showcased these metrics by applying them to DNN models trained on UK Biobank genotype data to predict standing height in a regression setting. Collectively, these benchmarking metrics illuminate the plausibility and reliability of DNN-based attribution algorithms.

## 2. Methods

**Fig 1:**
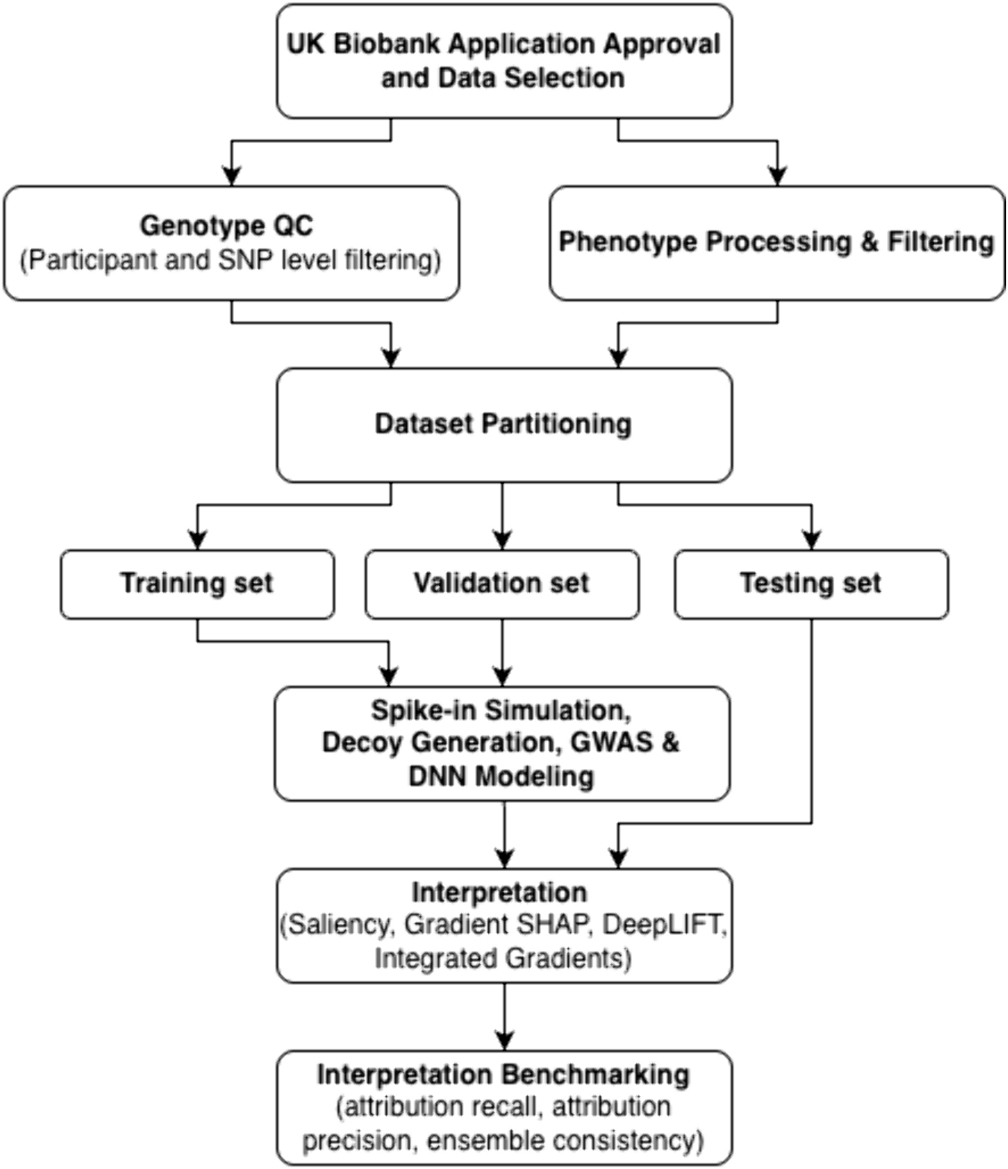
Overview of computational workflow employed for interpretation benchmarking. Following application approval and data selection, genotype quality control (QC) and phenotype processing and filtering were performed. The preprocessed data were then divided into training, validation, and testing sets. Spike-in SNPS and decoy genotypes were constructed along with running GWAS. DNN models were trained with the training set and optimized with the validation set. Association testing was performed by fitting a generalized linear model on the training set using PLINK to obtain GWAS results. Interpretation was then performed on the trained DNN model(s) with established interpretation algorithms including Saliency, Gradient SHAP, DeepLIFT, and Integrated Gradients. Finally, attribution recall, attribution precision, and attribution consistency were measured for the benchmarked interpretation methods.

An overview of the computational workflow used in this study is illustrated in Fig 1. This study utilized genotype and phenotype data from the UK Biobank (UKB), a large-scale biomedical database consisting of approximately 500 thousand participants aged 40–69 at recruitment [29]. Standing height was used as the phenotype of interest, as it is not only highly heritable, meaning a large fraction of the variation in height is attributable to genetic differences, but also polygenic, involving contributions from numerous genes [30,31]. Consequently, studies of height have offered insights into genetic influence on complex phenotypes and diseases, laying a foundation for exploring disease risk and trait variation more broadly [32,33].

### 2.1. Genotype and phenotype data preprocessing and quality control

Genotype calls for each of the 22 autosomal chromosomes from the UK Biobank were first merged into a single PLINK binary file set (bim, fam, bed files) containing approximately 500 thousand participants across approximately 800 thousand autosomal SNPs. This genotype dataset served as the starting point for subsequent quality control (QC) steps (S1 Table).

At the participant level, participants who identified as non-white British, had missing height labels, demonstrated sex incongruities (i.e., discordant self-reported and genetically inferred sex), or were related to other participants (third-degree relatives or closer) were excluded. At the SNP level, SNPs with excessive missingness, low minor allele frequency, or deviations from Hardy–Weinberg equilibrium were excluded. By applying these exclusions, we sought to minimize confounding factors and reduce noise from unreliable genotypes.

Standing height measurements were available for approximately 99.5% of UK Biobank participants. Since height is influenced by non-genetic factors such as age and sex, we first removed these effects by fitting a linear regression model using age and sex as inputs to predict height. The residuals from this model represented the portion of height not explained by age or sex [33]. The residuals were then standardized using z-score normalization for numerical stability. This transformation rescaled the adjusted heights to have a mean of zero and unit variance. The resulting standardized residuals were used as the phenotype labels for all downstream modeling and benchmarking procedures.

The remaining participants post-QC were stratified by sex and partitioned into training, validation, and test sets comprising 80%, 10%, and 10% of the preprocessed cohort, respectively. This approach ensured that sex distributions remained consistent across the training, validation and testing sets. The training set was used for phenotype adjustment, synthetic SNP generation, GWAS fitting, and DNN training. The validation set was used for tuning DNN hyperparameters. And the held-out testing set was used for model evaluation and interpretation benchmarking.

### 2.2. Genome-Wide Association Study (GWAS)

GWAS was performed by fitting a generalized linear model on the genotypes from the training set in addition to the adjusted height labels. This was done using the --glm option in PLINK 2.0 which executes per-SNP linear regression between the adjusted height labels and the genotype dosage matrix [34]. No covariates beyond those implicitly used for phenotype adjustment were included in the GWAS in effort to isolate the strictly genetic contribution to height. The resulting p-values from the linear association analysis were transformed to −log(p) to provide SNP-level importance scores, serving as a linear baseline reference model for interpretation benchmarking.

### 2.3. Deep neural network development

To implement the proposed DNN interpretation benchmarking tasks, we trained several DNNs to predict adjusted height from the preprocessed genotype data. The DNN architectures included three hidden layers of sizes 1000, 200, and 50, each with Rectified Linear Unit (ReLU) activations, batch normalization, and dropout (p = 0.5). The output layer was a single neuron with a linear activation for the regression task.

Training was performed using the Adam optimizer [35] with a learning rate of 1 × 10^-6^ (weight decay = 0.001), mean absolute error loss function with L2 regularization (L2 factor = 0.001), and a mini-batch size of 128 over 100 epochs. Hyperparameters were manually optimized according to the predictive performance on the validation set assessed with the Pearson Correlation Coefficient between predicted height and actual adjusted height. The final model parameters used for model evaluation and interpretation benchmarking were stored during the epoch with the best validation Pearson Correlation Coefficient. As with the GWAS, no covariates were used for DNN training in effort to understand unconfounded genetic contributions.

These settings and hyperparameters remained consistent across the various training runs required for the proposed interpretation benchmarking methods. However, the model trained for the attribution precision benchmarking method included double the number of input neurons to account for decoy genotypes. The seed used for controlling the nature of the random shuffling of participants during training was varied amongst the ten different models in the ensemble to achieve subtle variability amongst the trained models.

In total, twelve unique DNN models were trained to perform the three downstream interpretation benchmarking tasks. For attribution recall, a DNN model was trained using the processed genotypes alongside 400 synthetic spike-in salient SNPs following specific effect type patterns. For attribution precision, a DNN model was trained using the true processed genotypes in addition to null decoy genotypes composed of an equal number of participants and SNPs (i.e., number of input units was double the number of SNPs). And, for ensemble consistency, ten independent DNN models were trained using the true processed genotypes.

### 2.4. Attribution methods

To evaluate the relative importance of SNPs from the trained models, multiple interpretation algorithms were applied on the held-out testing set of approximately 33 thousand participants in order to estimate the contribution of each SNP on model predictions. These algorithms differ in their computational mechanisms and underlying theoretical principles, offering insights into how models arrive at a prediction. In this subsection, we describe the interpretation algorithms used for interpreting the trained DNN models across the proposed benchmarking tasks. All interpretation methods were implemented using Captum (v0.7.0), an open-source Python library developed for model interpretability with PyTorch-based DNN models [36]. Captum provides a unified API for applying a range of feature attribution methods and was used to ensure consistent and reproducible implementation. The following Captum modules were used: Saliency, Gradient SHAP, DeepLift, IntegratedGradients, and NoiseTunnel. All Captum algorithms were used with standardized configuration parameters to maintain comparability across interpretation methods. In particular, we consistently computed global attributions across all tested algorithms to scale the individual attributions with the input magnitude. The target output index was fixed to 0, corresponding to the single continuous prediction output of the network. Many interpretation methods quantify feature importance by comparing the model’s response at a given input *x* to its response at a baseline *x*′, effectively measuring how much each feature deviates from a reference value. The baseline is user-defined and important for ensuring that interpretation scores reflect meaningful contributions rather than artifacts. For this study, we consistently employed a mode genotype baseline where each SNP was set to the most frequently occurring allele in the training set to ensure feature importance scores were computed relative to a reference point that is representative of the population. This same baseline array was applied for all Captum methods, including those requiring an explicit baseline input.

We also evaluated each interpretation method in the presence of SmoothGrad (Captum’s NoiseTunnel module), a technique that averages attributions over multiple noisy perturbations of the input [37]. SmoothGrad mitigates the effects of sharp gradients or discontinuities in the learned function by applying Gaussian noise into the input. In our experiments, each tested interpretation algorithm was evaluated both in its standard form and in combination with SmoothGrad, enabling a direct assessment of whether noise-averaging improves interpretation performance across the proposed benchmarking metrics.

#### 2.4.1. Saliency.

Saliency estimates the importance of each input feature by computing the gradient of the model’s output with respect to the input [7]. This approach provides a first-order approximation to small perturbations, assuming local linearity. For a trained DNN, *f*(*X*), predicting an outcome *y* from input features X = (*x*_1_, *x*_2_, …, *x*_*d*_), the saliency score for the *j*-th feature is given by:


$$A_{j}=\frac{\partial f(X)}{\partial x_{j}}$$

where the partial derivative of f with respect to *x*_*j*_ represents the gradient of the model output with respect to the input *x*_*j*_. This method highlights the most sensitive variants in the model’s decision function but may be susceptible to local variations in gradient magnitudes.

#### 2.4.2. Gradient SHAP.

Gradient SHAP extends gradient-based interpretation by incorporating elements of Shapley values from cooperative game theory [8]. It approximates Shapley values by sampling from the baseline distribution and computing expected gradients along interpolated paths between the baselines and inputs. Specifically, for a model *f*, an input instance *x*, and a baseline distribution p(*x*′), the attribution for feature *j* is estimated as:


$$A_{j}={\text{E}}_{x^{\prime }\sim \text{p}\left({\text{x}}^{\prime }\right)}\left[\left(x_{j}-{\text{x}}_{\text{j}}^{\prime }\right)\cdot \frac{\partial \text{f}(\text{x})}{\partial x_{j}}\right]$$

where $x^{\prime }$ denotes a baseline sampled from p(*x*′), and the term (*x*_*j *_*− x*′_*j*_) corresponds to the difference between the input and the sampled baseline for feature *j*. The expectation is estimated by Monte Carlo sampling, making the method computationally intensive but capable of capturing feature importance in the presence of noise. The overall goal is to reduce gradient noise and better approximate the marginal contributions of each feature.

#### 2.4.3. Deep learning important FeaTures (DeepLIFT).

DeepLIFT is a backpropagation-based method that attributes the contribution of each input feature by comparing the change in the model’s output, Δ*y* = f(*x)* *– f(x*′), to the change in that feature relative to a baseline instance, Δ*x* = *x*_* *_*− x*′ [10,11]. For a given SNP *j*, the DeepLIFT attribution is defined as:


$$A_{j}=C_{\Delta x_{j}\to \Delta y}$$

where *C*Δ*x_j _* → Δ*y* denotes the total contribution that the input difference Δ*x_j _* propagates through the network to produce the output difference Δ*y*. DeepLIFT preserves additive composition, ensuring that contributions sum to the total output difference.

#### 2.4.4. Integrated gradients.

Integrated Gradients attributes feature importance by accumulating gradients along a path from a baseline input *x*′ to the input *x* [9]. For each feature *j*, the attribution is defined as the product of the input difference (*x*_*j *_*− x*′_*j*_) and the average gradient of the model output with respect to that feature, integrated over the path from the baseline *x*′ to input *x*:


$$A_{j}=\left(x_{j}-x_{j}^{\prime }\right)\times \int_{\alpha =\text{0}}^{\text{1}}\frac{\partial f\left(x^{\prime }+\alpha \left(x-x^{\prime }\right)\right)}{\partial x_{j}}\;d\alpha$$

where α is in the range [0, 1] and scales the interpolation between *x* and *x*′. The integral is approximated using a Riemann sum over evenly spaced points along the path. This approach avoids the local sensitivity of methods like Saliency, instead capturing the global effect of each input feature by averaging gradients.

#### 2.4.5. Aggregated interpretation scores.

Each of the methods described in Sections 2.4.1 – 2.4.4 were applied to the trained DNN models to generate importance scores for each participant on the testing set. The instance-wise importance scores were aggregated into model-wise importance scores by averaging the magnitude of the attributions across all attributed participants (i.e., those in the test set):


$$S_{j}=\frac{\text{1}}{N}\sum_{i=\text{1}}^{N}\left|A_{i,j}\right|$$

where *A*_*i,j*_ is the interpretation score assigned to SNP *j* for participant *i* for any given interpretation algorithm, and *N* is the total number of samples in the testing set. The absolute value ensures that both positive and negative influences are considered equally. These model-wise importance scores were then used for benchmarking interpretation methods as described in Section 2.5.

### 2.5. Benchmarking feature importance

Three proposed interpretation benchmark methods were defined to compare and evaluate the various DNN interpretation algorithms: (i) attribution recall, (ii) attribution precision, and (iii) ensemble consistency. The input for each benchmarking metric consisted of the model-wise importance scores described in Section 2.4.5.

#### 2.5.1. Attribution recall.

To evaluate the ability of DNN interpretation methods to recover known causal SNPs, attribution recall was defined using controlled synthetic spike-in SNPs as the ground-truth associations. Recall was defined as the proportion of synthetic SNPs of a given effect pattern (additive, dominant, recessive, epistatic) that appear among the k most highly attributed SNPs identified by a DNN interpretation method:


$$\text{Attribution Recal}{\text{l}}_{\text{k}}^{\left(\text{effect}\right)}=\frac{\left|\;\text{Top}{\text{K}}_{\text{DNN}}\;\cap \;\text{Syntheti}{\text{c}}_{\text{effect}}\;\right|}{\left|\;\text{Syntheti}{\text{c}}_{\text{effect}}\;\right|},$$

where $\text{Syntheti}{\text{c}}_{\text{effect}}$ represents the set of spike-in SNPs generated under a specific effect pattern and $\text{Top}{\text{K}}_{\text{DNN}}$ represents the k most highly attributed SNPs identified by a given algorithm. Thus, recall quantifies the fraction of truly associated synthetic SNPs that are accurately prioritized by a given interpretation method.

To create the ground-truth features used for recall benchmarking, we simulated 400 synthetic spike-in SNPs, evenly divided among effect types (i.e., 100 additive, 100 dominant, 100 recessive, and 100 epistatic). The epistatic set was constructed from 50 interacting pairs. These synthetic loci were then merged with the real genotypes, yielding an input matrix whose feature count equaled the sum of real and synthetic SNPs.

Each synthetic SNP was generated under Hardy-Weinberg equilibrium with a target MAF sampled from the empirical distribution of real SNPs. The standardized phenotype vector y (mean 0, variance 1) served as the correlation target for synthetic SNP generation. For each desired correlation coefficient ρ, a latent Gaussian variable was defined as


$$z=\rho y+\sqrt{\text{1}-\rho^{\text{2}}}\;e,$$

where e is an independent standard normal random vector. This formulation constructs a latent variable z whose correlation with y equals the target ρ, ensuring that the simulated SNPs exhibit controlled and realistic effect sizes while maintaining random residual variation.

The latent variable z was discretized into hard-call genotypes g∈{0,1,2} by applying MAF-specific thresholds chosen so that the expected genotype frequencies satisfied


$$P(g=\text{0})=(\text{1}-p{)}^{\text{2}},\;P(g=\text{1})=\text{2}p(\text{1}-p),\;P(g=\text{2})=p^{\text{2}}$$

where p is the MAF and P(g) represents the probability of observing genotype g. This ensured the simulated SNPs followed the expected genotype frequency distribution under Hardy-Weinberg equilibrium.

Additive SNPs were generated from this dosage variable, retaining loci with approximately linear genotype-phenotype relationships. Dominant SNPs were generated so that the carrier indicator C=1[g>0] was correlated with y, enforcing the pattern m1 ≈m2 > m0 where $m_{i}$ denotes the mean phenotype for genotype class i. Recessive SNPs were constructed such that the homozygous-alternate indicator R=1[g=2] was correlated with y, yielding m0 ≈m1 < m2. Epistatic pairs $\left(g_{A},g_{B}\right)$ were produced so that marginal correlations with y were near zero but the interaction term


$$H=\left(g_{A}-\text{2}p_{A}\right)\left(g_{B}-\text{2}p_{B}\right)$$

was correlated with the phenotype y at a target $\rho_{\text{epi}}$. Here, $p_{A}$ and $p_{B}$ denote the MAFs of the interacting SNPs. A line search procedure over a dependency parameter α∈[0, 0.95] adjusted $g_{B}$ such that the observed correlation of H with y approximated $\rho_{\text{epi}}$, while the marginal effect correlations remained below a small cap (|r|<0.01).

Target genotype-phenotype correlation magnitudes were drawn uniformly within the range of ρ∈[0.018, 0.070], with effect signs assigned at random. MAFs were sampled in the range of [0.05, 0.20]. Minimum expected counts of homozygous-alternate samples were enforced to be at least 1200 for recessive SNPs while dominant carrier fractions were constrained in the range of 10%-14%.

#### 2.5.2. Attribution precision.

To evaluate the specificity of DNN attribution methods in distinguishing biologically meaningful signals from noise, attribution precision was introduced. This metric quantifies the proportion of likely phenotype-associated real SNPs among the set of top-K most highly attributed real SNPs identified by an interpretation approach.

**Fig 2:**
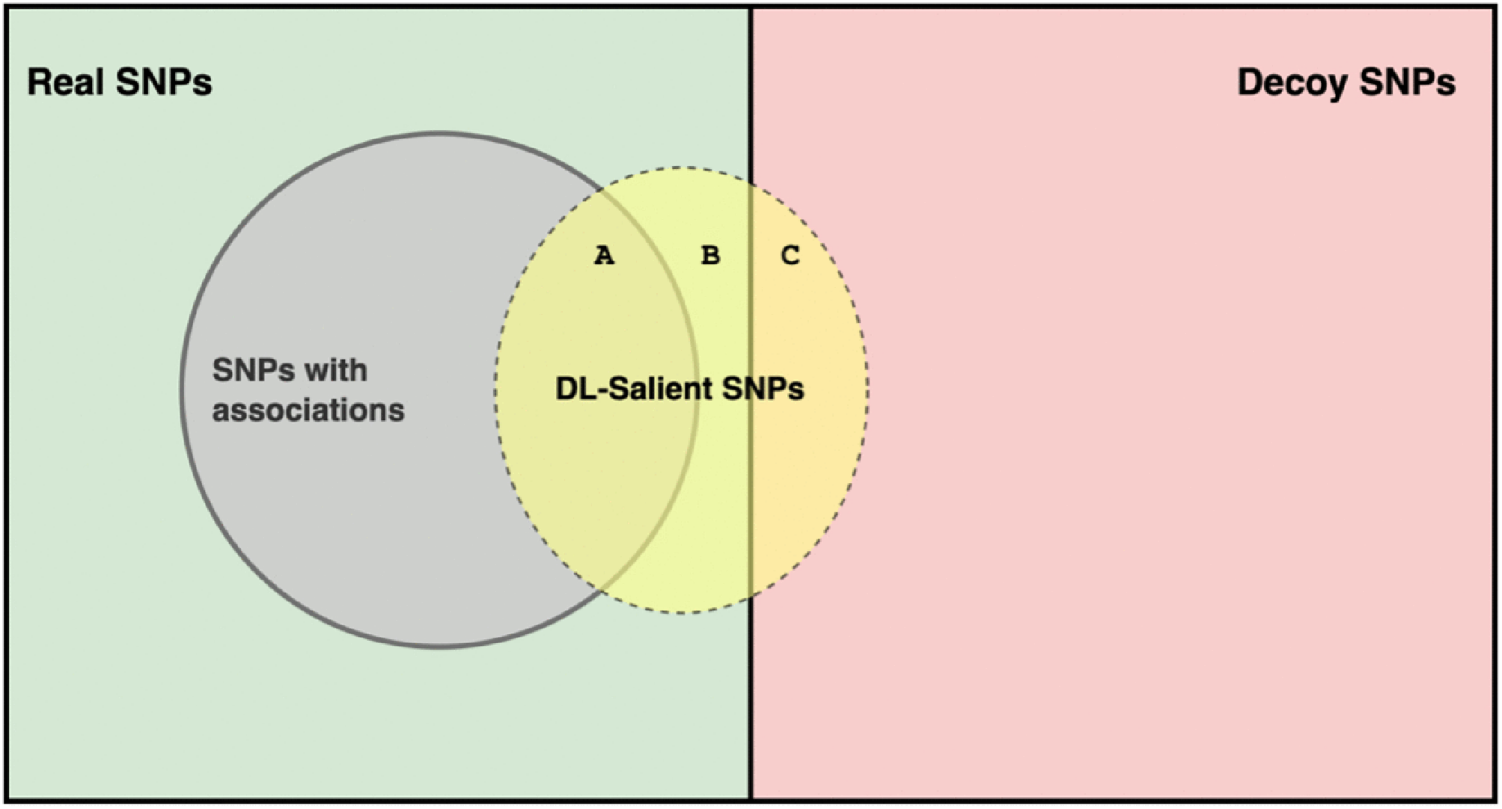
Illustration of the attribution precision metric. The full set of features consists of real SNPs (left, green) and decoy SNPs (right, red). Within the set of real SNPs, a small subset is truly associated with the phenotype (grey circle; SNPs with associations). A DNN interpretation method identifies a set of top-K SNPs (yellow oval; DL-Salient SNPs), containing three subsets: A (truly associated real SNPs), B (real SNPs lacking true association), and C (decoy SNPs). Since sets B and C are assumed to be comparable in size, the number of decoy SNPs in the top-K most highly attributed SNPs (C) is used as an estimate of the number of real SNPs lacking true association (B), enabling the calculation of attribution precision as 1 − (|*C*|/ |*A *+ *B*|).


The attribution precision metric illustrated in Fig 2 depicts the complete set of features comprising real SNPs and an equal number of synthetic decoy SNPs. Within the real SNPs, only a subset is truly associated with the phenotype. The DNN interpretation method identifies a subset of top-K salient SNPs that includes three categories: A (real SNPs with true phenotype association), B (real SNPs without phenotype association), and C (decoy SNPs). Because the true status of A and B is unknown, the number of real SNPs lacking true association (B) is approximated by the number of decoy SNPs (C). This approximation enables estimation of attribution precision as:

$${\text{Attribution Precision}}_{k}=\text{1}-\frac{\left|\text{TopK}(\text{Decoy})\right|}{\left|\text{TopK}(\text{Real})\right|}$$

where |TopK(Decoy)| is the number of decoys among the k most highly attributed SNPs and |TopK(Real)| is the number of real SNPs in the same set. The precision estimate reflects the proportion of real SNPs that are not equivalent to decoys and are thus likely to be biologically informative.

Decoy SNPs were generated by permuting the sample indices within each realized subset or chunk of the genotype, preserving per-variant allele frequency distributions while disrupting the genotype-phenotype correspondence. For each genotype subset loaded, a deterministic and chunk-aware random seed was assigned to produce a unique permutation of sample indices. This permutation mapping was reused across epochs within a given data split to maintain consistency, while distinct seeds were applied to the training, validation, and test sets to ensure independence. The resulting decoy matrix contained the same number of features as the real genotype matrix, and the two were concatenated along the feature dimension to form an expanded input matrix with twice as many SNP features as the original genotypes. Pseudocode describing this decoy generation procedure, as implemented in the DNN data loader module, is provided in S1 Protocol. This design ensured that the decoy genotypes were statistically equivalent to the real genotypes in their marginal distributions yet uninformative with respect to the phenotype. The decoy permutation was applied directly in the PyTorch and Dask-based [38] iterable dataset module that was used for streaming data during training, allowing each genotype chunk to be realized in memory alongside the corresponding decoy features without additional I/O overhead.

While our permutation-based decoy design provides a computationally efficient null baseline for estimating attribution precision, alternative approaches based on knockoff variables have been developed to contribute synthetic features that preserve the joint correlation structure of the original data while remaining statistically independent of the outcome [14,15]. Knockoff-based feature generation offers a principled way to control false discovery rates and could, in principle, yield more correlation-aware null baselines for attribution benchmarking, albeit at higher computational cost for biobank-scale genotype matrices.

To compute attribution precision, a DNN model was first trained on the expanded genotype matrix containing equal numbers of real and decoy SNPs. Model-wise attribution scores were then computed on the held-out test set, likewise balanced between real and decoy SNPs. The combined real-and-decoy attribution rankings were used to define each method’s top-K SNP set (i.e., the K highest ranked features). For each choice of K, attribution precision was calculated as the fraction of real SNPs estimated to be associated with height among that top-K set, thereby quantifying how effectively each approach prioritized true phenotype-associated signals over noise.

#### 2.5.3. Ensemble consistency.

To evaluate the stability of feature attribution across independently trained DNN models, we computed ensemble consistency by measuring the variability of SNP-level importance scores across an ensemble of independently trained DNN models (*M* = 10). Each model in the ensemble was trained using identical architecture and hyperparameters but with different random initializations and shuffled training data, ensuring slight variability in learned weights and convergence dynamics. For each interpretation algorithm, the absolute attribution score for SNP j and model m, denoted $S_{m,j}$, was computed across each ensemble member. To obtain a scale-free measure of cross-model variability, the relative standard deviation (RSD) for each algorithm and SNP was computed as the across-ensemble standard deviation ($\sigma_{j}$) of its attribution magnitudes divided by its across-ensemble mean ($\mu_{j}$):


$$RSD_{j}=\frac{\sigma_{j}}{\mu_{j}}$$

This normalization expresses the variability of each SNPs importance relative to its typical magnitude, providing a unitless measure of attribution stability that is comparable across algorithms with different scaling conventions.

For each algorithm, the distribution of ${\text{RSD}}_{j}$ values across all attributed SNPs was summarized by its median, as a measure of central tendency, and by the median absolute deviation (MAD) to provide a non-parametric estimate of across-ensemble dispersion:


$$\text{MAD}=\text{median}\left(\left|{\text{RSD}}_{j}-\text{median}\left(\text{RSD}\right)\right|\right)$$

MAD was then multiplied by a scaling factor of 1.4826 to make it comparable to the standard deviation under a normal distribution. With this formulation, lower median RSD and scaled MAD indicate improved ensemble consistency, implying that the attribution algorithm assigns similar importance to each SNP across independently trained models.

### 2.6. Composite score

A composite score was used to integrate recall, precision, and ensemble consistency into a single quantitative measure. We chose to evaluate recall and precision according to the top 1% threshold, representing the most confident attributions for each method and corresponding to a stringent interpretability view where only the highest-ranking features are considered. For recall, we combined the individual effect-type (additive, dominant, recessive, and epistatic) recall scores into a single overall recall using the micro-average, calculated as the total number of true positives across all effect type categories, or the number of simulated SNPs ranked within the top 1%, divided by the total number of true positives plus false negatives across those categories, or the total number of synthetic SNPs attributed. To ensure that all metrics were expressed on a comparable [0, 1] scale with higher values indicating better performance, ensemble consistency scores (i.e., the median SNP-wise RSD across independently trained ensemble members) were transformed using a half-life transformation:


$$S={\text{2}}^{-x/\tau },$$

where x is the consistency score (median RSD) and τ is a half-life constant that defines the threshold at which the score is halved. We set τ to the median RSD score across methods to provide a meaningful and interpretable scale. This mapping converts *lower-is-better* variability into unitless stability scores in the range of [0, 1], with every increment of τ halving the stability score. We then used the geometric mean across recall, precision, and transformed ensemble consistency to reward methods that perform consistently well across all three metrics.

## 3. Results

### 3.1. Attribution recall

Attribution recall quantified how effectively each interpretation method recovered synthetic SNPs with known causal effects that were embedded into the empirical genotypes. This metric directly measured the sensitivity of each interpretation method to additive and non-additive genetic architectures, including dominant, recessive, and epistatic effects. The ground-truth set included 100 additive, 100 dominant, 100 recessive, and 100 epistatic SNPs (from 50 interacting epistatic pairs) generated under Hardy-Weinberg equilibrium, as described in Section 2.5.1.

Recall scores for the top 1%, 5% and 10% of the most highly attributed SNPs for each algorithm, with and without SmoothGrad, are shown in Table 1. Results for additional thresholds (Top 2%, 3%, and 20%) are provided in S2 Table to illustrate findings at intermediate and broader cutoffs. 

**Table 1:** Attribution recall for DNN attribution algorithms and the linear GWAS model across the top 10%, 5%, and 1% of SNPs ranked by attribution magnitude.

Top %*	K	Algorithm	Smoothing**	Attribution recall^†^			
				Additive	Dominant	Recessive	Epistatic
1%	5530	Saliency	No	1.00	0.99	0.15	0.20
			Yes	1.00	0.99	0.16	0.20
		Gradient SHAP	No	1.00	0.40	0.01	0.20
			Yes	1.00	0.99	0.24	0.21
		DeepLIFT	No	1.00	0.39	0.01	0.20
			Yes	1.00	0.99	0.24	0.21
		Integrated Gradients	No	1.00	0.39	0.01	0.20
			Yes	1.00	0.99	0.24	0.21
		GWAS	No	1.00	0.83	0.00	0.00
5%	27650	Saliency	No	1.00	1.00	0.47	0.23
			Yes	1.00	1.00	0.47	0.23
		Gradient SHAP	No	1.00	0.95	0.35	0.25
			Yes	1.00	1.00	0.48	0.27
		DeepLIFT	No	1.00	0.94	0.35	0.25
			Yes	1.00	1.00	0.48	0.27
		Integrated Gradients	No	1.00	0.95	0.35	0.25
			Yes	1.00	1.00	0.48	0.27
		GWAS	No	1.00	1.00	0.16	0.00
10%	55300	Saliency	No	1.00	1.00	0.56	0.28
			Yes	1.00	1.00	0.55	0.28
		Gradient SHAP	No	1.00	1.00	0.44	0.34
			Yes	1.00	1.00	0.56	0.34
		DeepLIFT	No	1.00	0.99	0.44	0.35
			Yes	1.00	1.00	0.55	0.34
		Integrated Gradients	No	1.00	1.00	0.44	0.34
			Yes	1.00	1.00	0.56	0.34
		GWAS	No	1.00	1.00	0.44	0.02

* *Top %* indicates the percentage of most highly attributed SNPs and corresponds to the number of SNPs denoted in the column labeled *K*.

** Smoothing column denotes whether SmoothGrad was applied (no indicates the method was evaluated in the absence of SmoothGrad; Yes indicates the method was evaluated in the presence of SmoothGrad).

^†^Attribution recall is subdivided into four columns: *Additive* (synthetic additive effect recall), *Dominant* (synthetic dominant effect recall) *Recessive* (synthetic recessive effect recall), *Epistatic* (or synthetic epistatic effect recall).

All algorithms achieved perfect additive-effect recall across all tested thresholds. For dominant effects, all methods achieved high recall scores, but differences emerged across thresholds. Algorithms incorporating SmoothGrad consistently provided the highest recall for dominant effects. At the 10% threshold, recall approached 1.0 for all methods. At stricter thresholds (5% and 1%), SmoothGrad variations maintained near-perfect dominant-effect recall (0.99-1.00), whereas non-smoothed Gradient SHAP, DeepLIFT, and Integrated Gradients dominant-effect recall degraded sharply to 0.39-0.40 at the top 1% cutoff. GWAS achieved intermediate dominant-effect recall of 0.83 at 1%, which was inferior to the noise-averaged DNN attribution recall at 1% but superior to the non-noise-averaged DNN attribution recall at 1%.

For recessive effects, all DNN interpretation algorithms achieved substantially higher recall than GWAS, with SmoothGrad variants providing the largest gains at strict thresholds. At the 1% cutoff, recall was 0.24 for the smoothed versions of Gradient SHAP, DeepLIFT, and Integrated Gradients, compared with 0.01 for their non-smoothed counterparts and 0.00 for GWAS. Saliency and Saliency with SmoothGrad achieved recall values of 0.15 and 0.16, respectively. At the 5% threshold, recall reached approximately 0.47-0.48 for all SmoothGrad variants and 0.35-0.47 for non-smoothed DNN algorithms, compared with 0.16 for GWAS. At the 10% cutoff, recall values converged near 0.55-0.56 for the smoothed methods and 0.44-0.56 for the non-smoothed versions, while GWAS achieved 0.44. At the broadest 20% threshold, recall further increased to approximately 0.72-0.74 for the SmoothGrad algorithms and 0.66-0.69 for their non-smoothed counterparts, with GWAS reaching 0.69. Overall, SmoothGrad improved the recessive effect sensitivity of Gradient SHAP, DeepLIFT, and Integrated Gradients across most thresholds, whereas the Saliency method exhibited lower recall at the most stringent cutoffs but comparable performance at moderate and broad thresholds.

For epistatic effects, both smoothed and non-smoothed DNN attribution methods captured measurable interaction signals that the linear GWAS baseline almost entirely missed. At the 1% cutoff, recall was approximately 0.20-0.21 for all DNN algorithms, regardless of smoothing, while GWAS achieved 0.0. At the 5% threshold, recall reached approximately 0.23-0.27 for the DNN algorithms, with minimal difference between smoothed and non-smoothed variations, whereas GWAS remained at 0.0. By the 10% threshold, recall further increased to 0.34-0.35 for all DNN methods and 0.02 for GWAS. At the 20% threshold, recall reached 0.43-0.51 for the DNN algorithms, with minor, non-systematic differences between smoothed and non-smoothed versions, while GWAS achieved only 0.07. Overall, both smoothed and non-smoothed DNN attributions recovered a substantial fraction of interaction-driven associations that were undetectable by the linear model, with SmoothGrad providing no consistent advantage but maintaining comparable sensitivity across thresholds.

**Fig 3:**
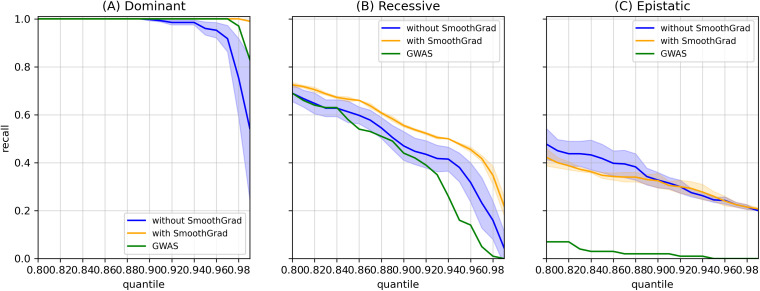
Mean attribution recall across quantile thresholds for DNN interpretation methods and GWAS. Mean recall values are shown for DNN attribution methods with (orange) and without (blue) SmoothGrad, compared with the GWAS baseline (green). Shaded regions represent the mean ± 1 standard deviation across replicates. **(A)** Dominant-effect recall. Smoothed DNN attribution methods achieved consistently higher recall than both non-smoothed variants and GWAS for dominant synthetic variants. **(B)** Recessive-effect recall. A similar trend was observed for recessive variants, where smoothed methods maintained greater sensitivity across thresholds. **(C)** Epistatic-effect recall. Both smoothed and non-smoothed DNN methods recovered measurable epistatic associations, substantially outperforming GWAS, which exhibited near-zero recall.

Fig 3 shows these relationships across quantile thresholds (or the 1 – Top %) for dominant (A), recessive (B), and epistatic (C) effects. SmoothGrad variations consistently achieved higher or comparable recall and lower variability compared to non-smoothed algorithms, while the GWAS was limited to additive and dominant effects. These results indicate that DNN-based interpretation methods, particularly when combined with SmoothGrad, are more sensitive than the linear GWAS baseline to both additive and non-additive genetic effects. Because all methods achieved perfect additive recall across all thresholds (recall = 1.0), additive recall curves were omitted from Fig 3.

### 3.2 Attribution precision

To assess each DNN attribution algorithm’s specificity in identifying phenotype-associated SNPs, we applied the proposed attribution precision benchmark metric described in Section 2.5.2. This metric evaluated the proportion of likely associated SNPs among the most highly attributed real SNPs by estimating the contribution of uninformative real SNPs using decoy genotypes. Attribution precision scores were generated across five top K thresholds, corresponding to the top 20%, 10%, 3%, 2%, 1% of most highly ranked SNP attributions (Table 2). At the strictest threshold (top 1%), all interpretation methods achieved relatively high precision scores between approximately 0.77 and 0.92, indicating that the most confident attributions were predominantly assigned to real SNPs.

**Table 2:** Attribution precision benchmarking results across the top 20%, 10%, 3%, 2%, and 1% of most highly attributed SNPs by attribution magnitude^†^.

Algorithm	Smoothing	Top 20%	Top 10%	Top 5%	Top 3%	Top 2%	Top 1%
**Saliency**	**No**	0.3553	0.5050	0.6451	0.7438	0.8146	0.9159
	**Yes**	0.3557	0.5055	0.6461	0.7436	0.8148	0.9165
**Gradient SHAP**	**No**	0.2363	0.3682	0.4971	0.5887	0.6589	0.7680
	**Yes**	0.3282	0.4681	0.5921	0.6814	0.7524	0.8493
**DeepLIFT**	**No**	0.2364	0.3678	0.4970	0.5897	0.6591	0.7681
	**Yes**	0.3286	0.4684	0.5932	0.6829	0.7507	0.8511
**Integrated Gradients**	**No**	0.2363	0.3682	0.4970	0.5887	0.6588	0.7678
	**Yes**	0.3282	0.4678	0.5917	0.6816	0.7519	0.8491

^†^Top X% represents the percentage of the most highly attributed SNPs considered for evaluation among all attributed SNPs. There were 234051 SNPs in the Top 20%, 117026 SNPs in the Top 10%, 35108 SNPs in the Top 3%, 23406 in the Top 2%, and 11703 SNPs in the Top 1%.

Performance diverged more noticeably at broader thresholds. Saliency-based methods demonstrated the highest overall specificity across all thresholds, with Saliency and Saliency with SmoothGrad achieving a precision of approximately 0.36 at the 20% threshold and approximately 0.51 at the 10% threshold. SmoothGrad generally improved precision across interpretation algorithms. For example, the SmoothGrad variations for DeepLIFT, Gradient SHAP and Integrated Gradients each achieved precision scores of approximately 0.47, 0.68 and 0.75 at the 10%, 3% and 2% thresholds, respectively, demonstrating improved selectivity for relevant features compared to their non-smoothed counterparts. In contrast, the non-smoothed variations of Gradient SHAP, DeepLIFT and Integrated Gradients exhibited reduced precision at more relaxed thresholds (approximately 0.38 at 10% and approximately 0.25 at 20%), indicating that a sizable fraction of their top-ranked attributions corresponded to SNPs lacking association. However, at stricter thresholds, all methods converged toward higher precision (approximately 0.76 at 1%).

**Fig 4:**
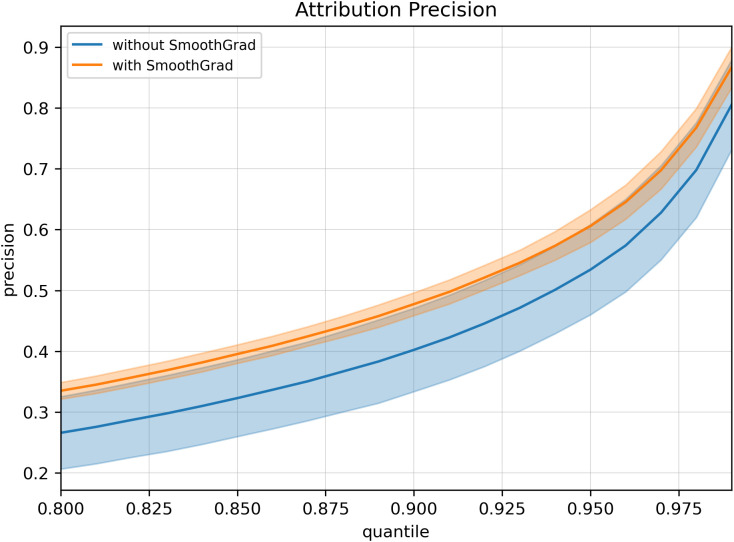
Average attribution precision across quantile thresholds for SmoothGrad and non-SmoothGrad variations. Lines represent the mean attribution precision across attribution algorithms (Saliency, DeepLIFT, Gradient SHAP, and Integrated Gradients) with (orange) and without (blue) SmoothGrad applied. Shaded regions denote ± 1 standard deviation across the algorithms included in each mean curve. Quantile values along the x-axis correspond to the top fraction of most highly attributed SNPs. Precision increased monotonically with stricter quantile thresholds, with SmoothGrad consistently improving attribution specificity relative to non-smoothed variants.

Attribution precision trends (Fig 4), shows the average precision of SmoothGrad and non-SmoothGrad variations across a continuous range of quantile thresholds from 0.80 to 0.99 (i.e., thresholds ranging from the top 20% to the top 1%). Precision increased monotonically as the quantile threshold became more stringent, reflecting that higher attribution magnitudes corresponded to SNPs with stronger genotype–phenotype associations. Across all quantiles, the smoothed variations achieved uniformly higher precision than their non-smoothed counterparts, confirming that gradient smoothing enhances the selectivity of attributions by reducing noise and stabilizing feature importance estimates. The widening separation between two curves at more relaxed thresholds further highlights the robustness of smoothing in isolating informative SNPs considered among the most highly ranked SNPs. Together, Table 2 and Fig 4 demonstrate that SmoothGrad consistently improves attribution specificity while maintaining similar high-confidence behavior at stricter thresholds.

### 3.3. Ensemble consistency

To assess the stability of feature attribution across independently trained DNN models, ensemble consistency was quantified using the relative standard deviation (RSD) of SNP-level attribution magnitudes across an ensemble of ten models. The RSD expresses the variability of each SNP’s importance relative to its mean attribution magnitude across ensemble members, providing a scale-free measure of interpretive stability. Lower RSD values indicate that a method assigns similar relative importance to each SNP across training runs, reflecting higher ensemble consistency.

Summary statistics of the RSD distributions for each interpretation algorithm are provided in Table 3. The median RSD values ranged from approximately 0.38 to 0.46, indicating moderate variability in attribution magnitudes across models. Among all algorithms, Saliency and Saliency with SmoothGrad exhibited the lowest median RSDs (0.38 and 0.41, respectively), suggesting that the relative importance assigned to SNPs by methods remained comparatively stable across ensemble members. In contrast, DeepLIFT, Integrated Gradients, and Gradient SHAP exhibited higher median RSDs (approximately 0.45), indicating somewhat greater sensitivity to model initialization and training variation.

**Table 3:** Ensemble consistency as model-wise SNP attribution variability.

Algorithm	Smoothing	Median RSD^†^	MAD*
**Saliency**	**No**	0.38294	0.11644
	**Yes**	0.41311	0.14581
**Gradient SHAP**	**No**	0.45137	0.15829
	**Yes**	0.42609	0.14209
**DeepLIFT**	**No**	0.45597	0.16199
	**Yes**	0.42586	0.14453
**Integrated Gradients**	**No**	0.45681	0.16379
	**Yes**	0.42971	0.14547

^†^Median RSD is the median relative standard deviation of SNP-wise attribution magnitudes across M = 10 ensemble members (as described in 2.5.3).

* MAD is the scaled median absolute deviation of RSDs to make it comparable to a standard deviation under a normal distribution (as described in 2.5.3).

Across all tested algorithms, the scaled MAD ranged from approximately 0.12 to 0.16, reflecting similar dispersion in RSD distributions among algorithms. SmoothGrad application produced mixed effects on consistency. For Saliency, RSD increased slightly (from 0.38 to 0.41), while for Gradient SHAP, DeepLIFT, and Integrated Gradients, the addition of SmoothGrad modestly reduced median RSDs (from approximately 0.45 to 0.43 for each). This suggests that noise-averaging via SmoothGrad may attenuate stochastic fluctuations in attribution magnitude for some methods, while having limited or opposite effects for others.

**Fig 5:**
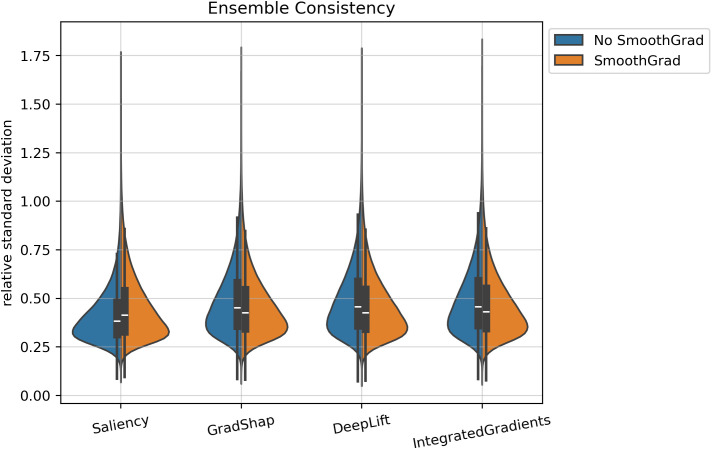
Distribution of SNP-wise relative standard deviations (RSD) of attribution magnitudes across ten ensemble members for each algorithm. For each method, the left (blue) half represents the non-SmoothGrad variant and the right (orange) half represents the SmoothGrad variant. Lower RSD values indicate higher ensemble consistency. Extended upper tails reflect outlier SNPs exhibiting greater attribution variability across models.

The full distribution of RSD values is shown in Fig 5, where each violin represents the spread of SNP-wise RSDs for smoothed and non-smoothed variations of each algorithm. Saliency methods displayed narrower distributions centered at lower RSDs, consistent with stronger cross-model stability. Gradient SHAP, DeepLIFT, and Integrated Gradients showed broader distributions and higher central tendencies, indicating more variability across independently trained models. Overall, all methods exhibited moderate ensemble consistency, with differences primarily reflecting the inherent stochastic sensitivity of each interpretability algorithm rather than large divergences in overall stability.

To further assess the influence of network architecture on ensemble consistency, supplementary ensembles were trained with two and four hidden layers, each comprising five independently trained models. The results, summarized in S3 Table, showed that median RSD and scaled MAD values followed the same general trends observed with the three-layer 10-model ensemble. For both architectures, Saliency and Saliency with SmoothGrad achieved the lowest median RSDs (approximately 0.29-0.32 for the two-layer and approximately 0.36-0.40 for the four-layer ensembles), indicating relatively stable attributions across ensemble members. Gradient SHAP, DeepLIFT, and Integrated Gradients exhibited higher median RSDs (approximately 0.33-0.34 for two layers and 0.42-0.44 for four layers) with slightly broader dispersion, consistent with modestly greater variability in attribution magnitudes. The application of SmoothGrad again produced mixed effects, with RSD increasing slightly for Saliency but modestly decreasing it for the other algorithms. Overall, ensemble consistency patterns remained similar across network depths, suggesting that attribution stability primarily reflects the intrinsic robustness of each interpretation algorithm rather than sensitivity to model architecture.

### 3.4. Composite score

The composite scores provided in Table 4, represented as the geometric mean across attribution recall, precision, and ensemble consistency, provide an aggregated and quantitative measure of DNN interpretation performance. Composite scores were computed using attribution recall and precision measured at the top 1% threshold of SNPs ranked by attribution magnitude. This threshold was chosen to emphasize high-confidence detection of signals, where interpretability methods are expected to recover truly causal features while minimizing false positives.

**Table 4:** Composite scores measured as the geometric mean across recall, precision and ensemble consistency to provide a single quantitative measure of interpretation performance.

Algorithm	Smoothing	Recall (Top 1%)^†^	Precision (Top 1%)^‡^	Ensemble consistency*	Composite score
**Saliency**	No	0.5850	0.9159	0.5378	0.6605
	Yes	0.5875	0.9165	0.5121	0.6509
**Gradient SHAP**	No	0.4025	0.7680	0.4813	0.5299
	Yes	0.6100	0.8493	0.5015	0.6381
**DeepLIFT**	No	0.4000	0.7681	0.4778	0.5275
	Yes	0.6100	0.8511	0.5017	0.6386
**Integrated Gradients**	No	0.4000	0.7678	0.4771	0.5272
	Yes	0.6100	0.8491	0.4985	0.6368

^†^Top 1% for recall corresponds to the top 5530 highest ranked SNPs per algorithm (sets composed of empirical genotypes and simulation genotypes).

^‡^Top 1% for precision corresponds to the top 11703 highest ranked SNPs per algorithm (sets composed of empirical genotypes and decoy genotypes).

* Ensemble consistency reported as the half-life normalized median of the relative standard deviation per algorithm.

The smoothed algorithm variations consistently outperformed the non-smoothed variations, with an average composite score of approximately 0.64 for the smoothed algorithms compared to approximately 0.56 for the non-smoothed algorithms. Among individual methods, Gradient SHAP, DeepLIFT, and Integrated Gradients showed the largest relative improvements when smoothing was applied, with composite scores increasing from approximately 0.53 to 0.64 for each. These gains were driven primarily by larger recall and precision scores at the top 1% quantile, while ensemble consistency remained comparable between smoothed and non-smoothed versions. In contrast, Saliency achieved similar composite scores with and without smoothing (approximately 0.66 vs. 0.65), suggesting that its baseline sensitivity and precision were already near-optimal for this architecture and dataset.

Overall, these results indicate that smoothing contributes to more reliable and generalizable interpretations, providing higher composite performance across methods. The improvement is especially notable for the advanced gradient-based attribution algorithms, where smoothing enhances both signal detection and precision without compromising ensemble consistency.

## 4. Discussion

This study introduced a benchmarking framework to evaluate DNN interpretation methods in large-scale genomic prediction tasks, using standing height from the UK Biobank as a model phenotype. The framework comprises three metrics including attribution recall, attribution precision, and ensemble consistency. Each quantifies a distinct dimension of interpretation performance, including sensitivity to established genetic associations, specificity in filtering out uninformative features, and stability of attributions across independent trainings.

Attribution recall assessed how effectively DNN interpretation methods recovered known causal SNPs by introducing synthetic spike-in variants with additive, dominant, recessive, and epistatic effects. This allowed recall to be measured against a controlled ground truth while preserving realistic allele frequency structure. Across effect types, all attribution methods achieved perfect recall for additive loci and consistently high recall for dominant loci. The greatest variation occurred for recessive and epistatic loci, where smoothing provided the most pronounced improvements. Non-smoothed gradient-based methods recovered few recessive loci at stringent thresholds, while their smoothed counterparts identified a substantially larger fraction and maintained higher recall across quantiles. Epistatic recall followed a similar trend, with smoothed methods achieving modest but consistent gains. The GWAS performed well for dominant loci but showed negligible recovery of recessive or epistatic variants, underscoring its inability to detect nonlinear interactions. Saliency recovered non-additive SNPs more effectively than GWAS but with less improvement from smoothing. These patterns demonstrate that DNNs can capture nonlinear structure missed by linear models and that smoothing stabilizes gradient signals sufficiently to improve recovery of interaction-driven effects.

Attribution precision quantified specificity by measuring the proportion of attributed SNPs that represented true rather than null or decoy signals. The decoy generation approach used in this framework was efficient as decoy genotypes were generated in an online fashion during training and interpretation. This design eliminated the need to store and load large auxiliary data files. Decoy SNPs were produced by permuting sample indices within each subset of the realized dosage matrix, preserving allele frequency distributions while disrupting genotype-phenotype correspondence. Precision increased monotonically across controlled thresholds and consistently benefited from smoothing across all attribution algorithms. Smoothed variations of Gradient SHAP, DeepLIFT, and Integrated Gradients achieved higher precision than their non-smoothed counterparts, indicating that noise-averaged gradients effectively suppress spurious attributions. Saliency achieved the highest precision overall but showed diminishing gains from smoothing. Together, these findings indicate that smoothed gradient-based methods provide a favorable balance of sensitivity and specificity, particularly for identifying true positive loci among top-ranked attributions.

Ensemble consistency measured the stability of attributions across independently trained models. Consistency was evaluated using the median RSD of SNP-wise attributions across ensemble members. RSD values indicated minimal to moderate cross-model variability, with Saliency providing the most stable attributions, as shown by lower RSDs relative to the other methods. Smoothing reduced variability for Gradient SHAP, DeepLIFT, and Integrated Gradients while slightly increasing variability for Saliency. Median absolute deviations were similar across methods and variations, consistent with overall moderate cross-model variability. The same stability ranking observed in the 10-model, three-layer ensemble was also reflected in two- and four-layer ensembles, indicating that attribution stability depends more on the interpretation algorithm than on network depth.

The composite score integrated recall, precision, and ensemble consistency into a single quantitative summary to capture the multidimensional nature of interpretability. Smoothed variants of Gradient SHAP, DeepLIFT, and Integrated Gradients achieved average composite scores near 0.64, outperforming their non-smoothed counterparts, which averaged around 0.53. Saliency achieved the highest composite score overall (approximately 0.66) with negligible change in performance with smoothing, as both the smoothed and non-smoothed versions performed nearly identically across recall, precision, and stability. This pattern illustrates that smoothing most strongly benefits the more advanced gradient-based attribution methods, where smoothing techniques can reduce noise and highlight consistent signal. In contrast, simpler gradient methods like Saliency already produce highly stable and high-achieving attributions. Overall, the composite results indicate that SmoothGrad enhances interpretation performance for the more advanced gradient-based algorithms such as Gradient SHAP, DeepLIFT, and Integrated Gradients. Notably, Saliency achieved the highest overall composite score, demonstrating that first-order gradient methods can provide highly accurate and robust attributions in large-scale genomic settings.

Several limitations should be considered in the context of this framework. Simulation-based ground truth SNPs provide a controlled benchmarking strategy but may not fully capture the breadth of genetic complexity, such as linkage disequilibrium, gene-environment interactions, or polygenic effects. While our decoy-based precision metric provides an effective and scalable null baseline, it does not intentionally reproduce the full joint structure of genomic correlations. Deep-learning adaptations of the knockoff framework, such as DeepPINK [39], could offer a correlation-preserving substitute for the current decoy-based null features applied in the attribution precision benchmark. Future work may focus on extending this framework to additional phenotypes and DNN architectures and integrating biological enrichment analyses to evaluate whether highly attributed loci correspond to known regulatory regions or disease-associated SNPs. While this study used standing height as a representative phenotype to demonstrate the framework, the proposed benchmarking procedures are phenotype-agnostic and apply equally to quantitative, binary and multiclass traits.

## Supporting information

S1 Table Participant and SNP level quality control criteria. (DOCX)

S2 Table Attribution recall for DNN attribution algorithms (with and without SmoothGrad) and the linear GWAS model across the top 2%, 3%, and 20% of SNPs ranked by attribution magnitude. (DOCX)

S3 Table Effect of network architecture on ensemble consistency across DNN interpretation algorithms. Each ensemble consisted of five independently trained models sharing the same optimization parameters and data splits but differing in the number of hidden layers (two, three, or four). Reported values summarize the median and standard deviation (×10⁻⁴) of SNP-level attribution variability across ensemble members for each interpretation algorithm, with and without SmoothGrad noise averaging. Results show that ensemble consistency remained stable across architectures: Gradient SHAP, DeepLIFT, and Integrated Gradients consistently exhibited the lowest variability (~0.4-0.5 × 10⁻⁴), whereas Saliency-based methods were most variable (~1.6 × 10⁻⁴).
(DOCX)

S1 Protocol Online construction of decoy genotypes. Pseudocode describing the in-memory procedure used to generate decoy SNP features during data streaming. For each genotype chunk loaded into memory, a deterministic row permutation is applied to create a decoy matrix that preserves per-SNP allele frequencies and within-chunk linkage structure while disrupting genotype–phenotype correspondence. Each chunk receives a fixed, split-specific random seed (decoy_seed + σ(S) + chunk_idx), ensuring reproducible yet independent decoys across epochs and data splits without requiring disk-based storage.
(TIF)
