# Supplementary material for: Benchmarking interpretability of deep learning for predictive genomics: Recall, precision, and variability of feature attribution
Source: PLoS Comput Biol. 2025 Dec 5;21(12):e1013784. doi: 10.1371/journal.pcbi.1013784 (PMC12680242; doi:10.1371/journal.pcbi.1013784)
Supplement: S1 Table — (DOCX) [file pcbi.1013784.s001.docx]

**S1 Table. Participant and SNP level quality control criteria.**

| **Filter dimension** | **Criterion** | **Description** | **Data source** |
| --- | --- | --- | --- |
| Participant | Ethnicity | Exclude non-white British ethnicity participants | Main dataset (FID 31) |
|  | Sex | Exclude participants with discordant sex (differing self-reported and genetic sex) | Main dataset (FID 31 & 22001) |
|  | Relatedness | Exclude related participants (3^rd^ degree relatives or closer) | Main dataset (FID 22020) |
|  | Sex chromosome aneuploidy | Exclude participants with an abnormal number of sex chromosomes | Main dataset (FID 22019) |
|  | Unavailable trait label | Exclude participants without phenotype (height) labels | Main dataset (FID 50) |
|  | Participant missingness  (--mind 0.1) | Exclude participants with >10% missing genotypes | Genotype Calls |
| SNP | SNP missingness  (--geno 0.1) | Exclude SNPs with >10% missing genotype calls | Genotype Calls |
|  | Hardy-Weinberg  (--hwe 1e-15) | Exclude SNPs that deviate from HWE  (p < 1x10^-15) | Genotype Calls |
|  | Minor allele frequency  (--maf 0.01) | Exclude SNPs with MAF <1% | Genotype Calls |
|  | Minor allele count  (--mac 100) | Exclude SNPs with minor allele count <100 | Genotype Calls |
