# Supplementary material for: Benchmarking interpretability of deep learning for predictive genomics: Recall, precision, and variability of feature attribution
Source: PLoS Comput Biol. 2025 Dec 5;21(12):e1013784. doi: 10.1371/journal.pcbi.1013784 (PMC12680242; doi:10.1371/journal.pcbi.1013784)
Supplement: S2 Table — (DOCX) [file pcbi.1013784.s002.docx]

**S2 Table. Attribution recall for DNN attribution algorithms (with and without SmoothGrad) and the linear GWAS model across the top 2%, 3%, and 20% of SNPs ranked by attribution magnitude.**

| **Top %*** | **K** | **Algorithm** | **Smoothing** | **Additive** | **Dominant** | **Recessive** | **Epistatic** |
| --- | --- | --- | --- | --- | --- | --- | --- |
| 2% | 11060 | Saliency | No | 1.00 | 1.00 | 0.28 | 0.20 |
|  |  |  | Yes | 1.00 | 1.00 | 0.29 | 0.20 |
|  |  | Gradient SHAP | No | 1.00 | 0.67 | 0.12 | 0.22 |
|  |  |  | Yes | 1.00 | 1.00 | 0.37 | 0.22 |
|  |  | DeepLIFT | No | 1.00 | 0.67 | 0.12 | 0.22 |
|  |  |  | Yes | 1.00 | 1.00 | 0.37 | 0.22 |
|  |  | Integrated Gradients | No | 1.00 | 0.67 | 0.12 | 0.22 |
|  |  |  | Yes | 1.00 | 1.00 | 0.36 | 0.22 |
|  |  | GWAS | No | 1.00 | 0.97 | 0.01 | 0.00 |
| 3% | 16590 | Saliency | No | 1.00 | 1.00 | 0.39 | 0.21 |
|  |  |  | Yes | 1.00 | 1.00 | 0.40 | 0.21 |
|  |  | Gradient SHAP | No | 1.00 | 0.89 | 0.18 | 0.23 |
|  |  |  | Yes | 1.00 | 1.00 | 0.43 | 0.23 |
|  |  | DeepLIFT | No | 1.00 | 0.89 | 0.18 | 0.23 |
|  |  |  | Yes | 1.00 | 1.00 | 0.42 | 0.23 |
|  |  | Integrated Gradients | No | 1.00 | 0.89 | 0.18 | 0.23 |
|  |  |  | Yes | 1.00 | 1.00 | 0.42 | 0.23 |
|  |  | GWAS | No | 1.00 | 1.00 | 0.05 | 0.00 |
| 20% | 110600 | Saliency | No | 1.00 | 1.00 | 0.74 | 0.38 |
|  |  |  | Yes | 1.00 | 1.00 | 0.74 | 0.37 |
|  |  | Gradient SHAP | No | 1.00 | 1.00 | 0.68 | 0.51 |
|  |  |  | Yes | 1.00 | 1.00 | 0.72 | 0.43 |
|  |  | DeepLIFT | No | 1.00 | 1.00 | 0.66 | 0.51 |
|  |  |  | Yes | 1.00 | 1.00 | 0.72 | 0.45 |
|  |  | Integrated Gradients | No | 1.00 | 1.00 | 0.68 | 0.51 |
|  |  |  | Yes | 1.00 | 1.00 | 0.72 | 0.44 |
|  |  | GWAS | No | 1.00 | 1.00 | 0.69 | 0.07 |

*Top % indicates the top fraction (as a percentage) of SNPs with the highest attribution scores assigned by each algorithm, which directly corresponds to the number of SNPs denoted in the column labeled K.
