# Supplementary material for: Benchmarking interpretability of deep learning for predictive genomics: Recall, precision, and variability of feature attribution
Source: PLoS Comput Biol. 2025 Dec 5;21(12):e1013784. doi: 10.1371/journal.pcbi.1013784 (PMC12680242; doi:10.1371/journal.pcbi.1013784)
Supplement: S3 Table — Each ensemble consisted of five independently trained models sharing the same optimization parameters and data splits but differing in the number of hidden layers (two, three, or four). Reported values summarize the median and standard deviation (×10⁻⁴) of SNP-level attribution variability across ensemble members for each interpretation algorithm, with and without SmoothGrad noise averaging. Results show that ensemble consistency remained stable across architectures: Gradient SHAP, DeepLIFT, and Integrated Gradients consistently exhibited the lowest variability (~0.4-0.5 × 10⁻⁴), whereas Saliency-based methods were most variable (~1.6 × 10⁻⁴). (DOCX) [file pcbi.1013784.s003.docx]

**S3 Table. Supplementary ensemble runs showing median† and scaled median absolute deviation†† of SNP-wise relative standard deviations (RSD) across five-model ensembles trained with two and four hidden layers alongside the 3-layer 10-model ensemble.**

| **Algorithm** | **Smoothing** | **10-model 3L*** | | **5-model 2L**** | | **5-model 4L***** | |
| --- | --- | --- | --- | --- | --- | --- | --- |
|  |  | **RSD Median** | **RSD MAD** | **RSD Median** | **RSD MAD** | **RSD Median** | **RSD MAD** |
| **Saliency** | **No** | 0.38294 | 0.11644 | 0.28968 | 0.16673 | 0.36118 | 0.19208 |
|  | **Yes** | 0.41311 | 0.14581 | 0.31763 | 0.19759 | 0.40023 | 0.22459 |
| **Gradient SHAP** | **No** | 0.45137 | 0.15829 | 0.33729 | 0.21002 | 0.43047 | 0.22276 |
|  | **Yes** | 0.42609 | 0.14209 | 0.32440 | 0.20289 | 0.41884 | 0.22015 |
| **DeepLIFT** | **No** | 0.45597 | 0.16199 | 0.33554 | 0.21178 | 0.44148 | 0.21662 |
|  | **Yes** | 0.42586 | 0.14453 | 0.32304 | 0.20337 | 0.42811 | 0.21377 |
| **Integrated Gradients** | **No** | 0.45681 | 0.16379 | 0.33937 | 0.21350 | 0.43337 | 0.22629 |
|  | **Yes** | 0.42971 | 0.14547 | 0.32609 | 0.20558 | 0.42022 | 0.22240 |

†Median RSD represents the median relative standard deviation across ensemble members.

††MAD quantifies dispersion of RSDs, as defined in Section 2.5.3.

* 10-model 3L denotes the ensemble of ten DNNs, each composed of three hidden layers.

** 5-model 2L denotes the ensemble of five DNNs, each composed of two hidden layers.

*** 5-model 4L denotes the ensemble of five DNNs, each composed of four hidden layers.
